# Calf Spleen Extractive Injection protects mice against cyclophosphamide-induced hematopoietic injury through G-CSF-mediated JAK2/STAT3 signaling

**DOI:** 10.1038/s41598-017-08970-3

**Published:** 2017-08-21

**Authors:** Wenqian Lu, Dongxu Jia, Shengshu An, Ming Mu, Xinan Qiao, Yan Liu, Xin Li, Di Wang

**Affiliations:** 1 School of Life Sciences, Jilin University, Changchun, 130012 China; 2 Zhuhai College of Jilin University, Jilin University, Zhuhai, 519000 China

## Abstract

Calf Spleen Extractive Injection (CSEI), extracted from the spleen of healthy cows (within 24 hours of birth), is a small-peptide-enriched extraction and often used as an ancillary agent in cancer therapy. This study evaluated the hematopoietic function of CSEI and its underlying mechanisms, principally in CHRF, K562 cells, BMNCs and a mouse model of cyclophosphamide (CTX)-induced hematopoietic suppression. CSEI promoted the proliferation and differentiation of CHRF and K562 cells, activated hematopoietic- and proliferation-related factors RSK1p90, ELK1 and c-Myc, and facilitated the expression of differentiation- and maturation-related transcription factors GATA-1, GATA-2. In the mice with hematopoietic suppression, 3 weeks of CSEI administration enhanced the bodyweights and thymus indices, suppressed the spleen indices and strongly elevated the production of HSPCs, neutrophils and B cells in bone marrow, ameliorated bone marrow cellularity, and regulated the ratio of peripheral blood cells. Proteome profiling combined with ELISA revealed that CSEI regulated the levels of cytokines, especially G-CSF and its related factors, in the spleen and plasma. Additional data revealed that CSEI promoted phosphorylation of STAT3, which was stimulated by G-CSF in both mice spleen and cultured BMNCs. Taken together, CSEI has the potential to improve hematopoietic function via the G-CSF-mediated JAK2/STAT3 signaling pathway.

## Introduction

The hematopoietic system, comprising the entire system of blood production, is composed of hematopoietic cells and organs, the latter including the bone marrow, lymph nodes, thymus, spleen and liver^1^. Bone marrow is the main source of hematopoietic progenitors and is where red blood cells, granulocytes, megakaryocytes, lymphocytes and monocytes are generated^2^. The spleen can recognize and destroy abnormal red blood cells, and also store blood cells and filters out the bacteria, foreign bodies, antigen–antibody complexes and other harmful substances in blood^3, 4^. The occurrence of infectious or hemolytic anemia triggers extramedullary hematopoiesis (EMH), leading to swelling of the hematopoietic organs^5^.

Hematopoiesis is not only regulated by the hematopoietic microenvironment, but also influenced by positive or negative hematopoietic regulatory factors including interleukins (ILs), colony-stimulating factors (CSFs) and chemokines^6^. Hematopoietic dysfunction including myelosuppression, hematopoietic inhibition and immunosuppression is observed in patients with malignant tumors receiving high-dose radiotherapy and chemotherapy^7–9^. Rebuilding the hematopoietic function and immune system is the primary issue in the adjuvant treatment of chemotherapy. Granulocyte colony-stimulating factor (G-CSF) has been used clinically as an auxiliary chemotherapeutic agent due to its functionality in reducing chemotherapy-induced infections in cases of nonlymphoid malignancies, curing neutropenia and mobilizing hematopoietic cells into peripheral blood through binding to cognate cell surface receptors. Previous studies have suggested that G-CSF successfully regulated the signal transducers and activators of transcription (STAT) signaling^10, 11^.

Calf spleen extractive injection (CSEI), extracted from the spleen of healthy cows (within 24 hours of birth), is a small-peptide-enriched extraction that has been listed in China under State Medical Permit No. H22026121. CSEI exerts a variety of physiological and pharmacological effects, and is commonly used as an ancillary agent to assist cancer patients with immune dysfunction in clinical care^12, 13^. In our group, we selectively induced apoptosis in human hepatocellular carcinoma cells via a reactive oxygen species (ROS)/mitogen-activated protein kinases (MAPKs)-dependent mitochondrial pathway^14^, and effectively improved immune function in CTX-induced immunosuppression related to the nuclear factor kappa-B (NF-κB) signaling pathway^15^. CSEI has been widely used clinically in the treatment of aplastic anemia and primary thrombocytopenia. In clinical trials, CSEI has significantly increased the generation of red blood cells, hemoglobin and platelets in patients with cancer anemia^16, 17^. However, no studies have reported on the protective effects of CSEI against hematopoietic injury in animal models or systematically investigated its molecular mechanisms.

To further address the multifunctional activities of CSEI in hematopoiesis, we analyzed its effects on the proliferation and differentiation on hematopoietic cells in K562 and CHRF cells and measured its protective activities in a mouse model of CTX-induced hematopoietic injury and in cultured bone marrow mononuclear cells (BMNCs). Combining the *in vitro* and *in vivo* data, possible mechanisms involving G-CSF-mediated Janus kinase 2 (JAK2) /STAT3 signaling were further explored.

## Results

### CSEI promoted proliferation and differentiation of the hematopoietic cells

**Figure 1:**
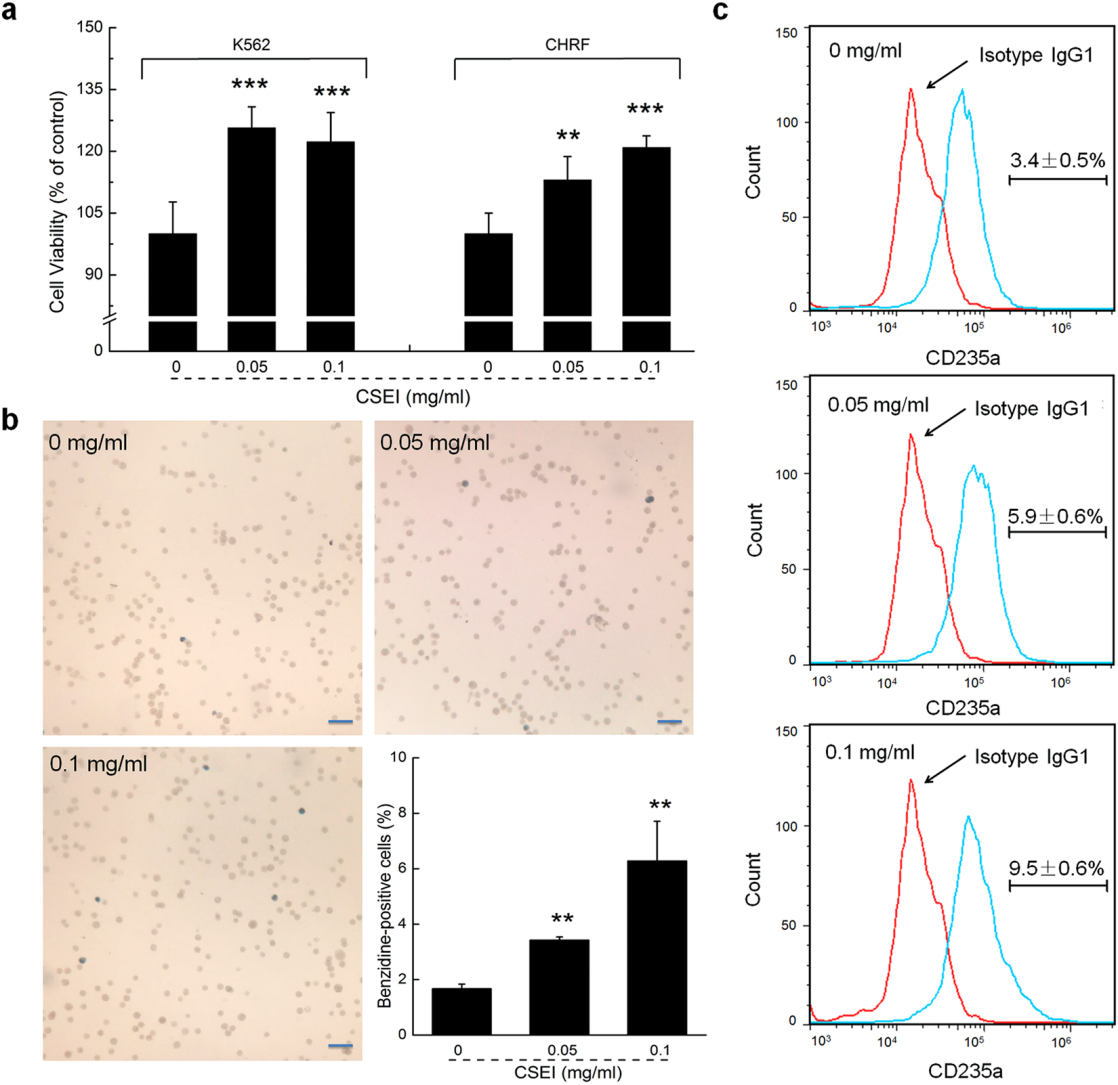
CSEI promoted proliferation and differentiation of CHRF and/or K562 cells. After a 24-h treatment of CSEI at doses of 0, 0.05 and 0.1 mg/ml, (**a**) cell viability of CHRF and K562 cells was detected by XTT assay, (**b**) the erythroid differentiation of K562 cells was analyzed by benzidine staining (10×, scale bar: 100 μm) and (**c**) the expression of glycophorin A (CD235a) in K562 cell line was analyzed by flow cytometry. Data are shown as the means ± S.E.M. (n = 6). **P < 0.01 and ***P < 0.001 versus 0 mg/ml CSEI-treated cells.

K562 cells, which resemble human erythroid and megakaryocytic progenitors, have been widely used in a human hematopoietic cell model to study the differentiation of erythrocytes and megakaryocytes^18–20^; CHRF cells resemble human megakaryoblasts. The XTT assay showed that CSEI significantly increased cell viability of the K562 and CHRF cells (*P* < 0.01; Fig. 1a). The erythroid differentiation in the K562 cells was determined by hemoglobinization and the expression of erythrocyte antigen glycophorin A (CD235a) via benzidine staining assay or flow cytometric assay. The proportion of benzidine-positive cells after 24 h of CSEI incubation increased to 6.3% from 1.6% (*P* < 0.01; Fig. 1b), whereas the expression of erythrocyte antigen glycophorin A in K562 cells was increased to 9.5% from 3.4% (Fig. 1c). Annexin V/PI staining indicated that CSEI failed to induce cell apoptosis of K562 and CHRF cells (Supplementary Fig. S1).

### CSEI regulated the expressions of proteins related to hematopoietic function in hematopoietic cells

**Figure 2:**
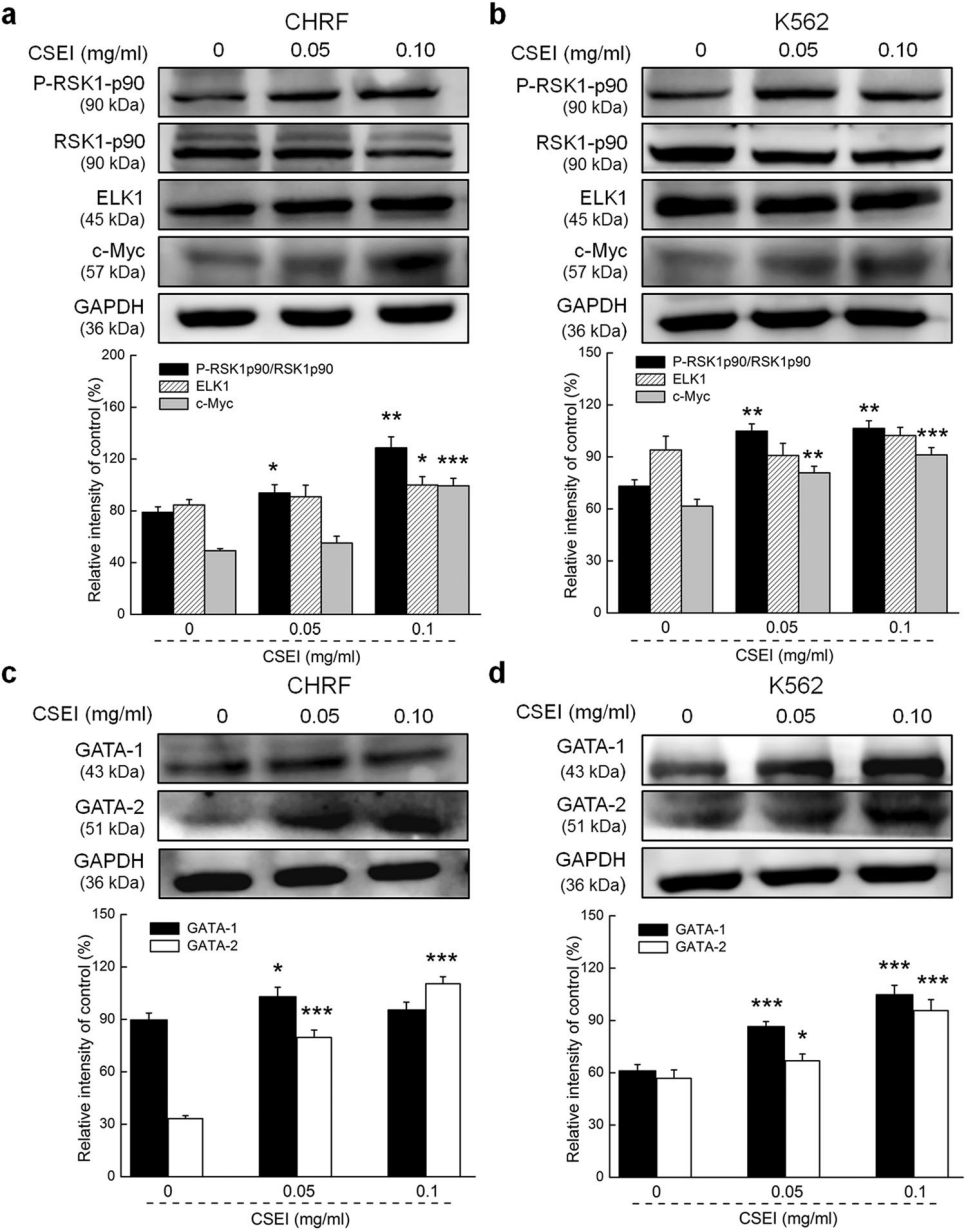
CSEI regulated the expressions of hematopoietic function related proteins in CHRF and K562 cells. After a 24-h CSEI (0, 0.05, 0.1 mg/ml) treatment, the protein expression levels of P-RSK1p90, ELK1 and c-Myc in CHRF cells (**a**) and K562 cells (**b**), full-length blots are presented in Supplementary Fig. S5 a, and the expression levels of GATA-1 and GATA-2 in the terminal mature CHRF cells (**c**) and differentiated K562 cells (**d**) were detected by western blotting, respectively, full-length blots are presented in Supplementary Fig. S5 (**b**). The quantitative data of these protein levels were normalized by their GAPDH expressions and expressed as a percentage of the corresponding relative intensity of control. The data are shown as the means ± S.E.M. (n = 6). *P < 0.05, **P < 0.01 and ***P < 0.001 versus 0 mg/ml CSEI-treated cells.

p90 ribosomal S6 kinases (RSK1p90), c-Myc and ETS transcription factor (ELK1), which have been recognized as endonuclear transcription factors related to cell growth^21^, were detected in the CHRF and K562 cells. CHRF and K562 cells incubated for 24 h in CSEI showed enhanced expression of phosphor(P)-RSK1p90 (by ≥ 45%), c-Myc (by ≥ 50%) and ELK1 (by ≥ 10%) (*P* < 0.05; Fig. 2a and b).

GATA-binding factor 1 (GATA-1) and GATA-binding factor 2 (GATA-2), factors related to differentiation and megakaryocyte maturation in the hematopoietic progenitor cells, were analyzed via western blotting. Compared with the control cells, CSEI facilitated the expression of GATA-1 and GATA-2 in the CHRF and K562 cells after 24 h of incubation (*P* < 0.05; Fig. 2c and d).

### Effects of CSEI on bodyweights and organ indices in CTX-injected mice with hematopoietic injury

The bodyweights of the mice were recorded during modeling and drug administration. After 3 weeks of treatment with CSEI or Tα1, the mice were euthanized, the spleens and thymus tissues were collected and weighed immediately, and organ indices were calculated. CTX significantly decreased the bodyweight of the mice, and this effect was reversed by both CSEI and Tα1 treatment (*P* < 0.05; Table 1). In addition, CTX resulted in splenomegaly and thymus reduction in the mice; in contrast, both CSEI and Tα1 reversed these abnormalities to normal levels (*P* < 0.05; Table 1).

**Table 1:** CSEI regulated bodyweights and organ indices in CTX-injected mice with hematopoietic injury.

	Day	CTRL	Model	Tα1 (0.24 mg/kg)	CSEI (mg/kg)		
					2.25	4.50	9.00
Bodyweights (g)	1^st^	19.5 ± 0.4	19.8 ± 0.6	20.0 ± 0.4	19.8 ± 0.4	19.6 ± 0.4	19.5 ± 0.4
	4^th^	20.4 ± 0.4	17.7 ± 0.2^##^	18.1 ± 0.2	18.3 ± 0.2*	17.8 ± 0.2	17.6 ± 0.2
	11^th^	20.6 ± 0.5	17.2 ± 0.3^##^	18.3 ± 0.2*	19.2 ± 0.2**	18.8 ± 0.4*	18.6 ± 0.3*
	18^th^	20.3 ± 0.5	18.1 ± 0.4^#^	18.8 ± 0.3	19.6 ± 0.2*	19.7 ± 0.5*	20.3 ± 0.6**
	25^th^	20.8 ± 0.7	17.8 ± 0.5^##^	19.1 ± 0.7	20.2 ± 0.5**	21.8 ± 0.7**	20.1 ± 0.7*
Organ index (%)	Spleen index	0.57 ± 0.05	0.80 ± 0.06^#^	0.60 ± 0.03**	0.62 ± 0.03*	0.60 ± 0.01*	0.69 ± 0.02
	Thymus index	0.15 ± 0.02	0.09 ± 0.02^#^	0.15 ± 0.01*	0.19 ± 0.02**	0.21 ± 0.02**	0.11 ± 0.01

100 mg/kg of CTX was intraperitoneally injected to mice except controls for three days. Then Tα1 and CSEI were treated for another 21 days. The bodyweights of mice were monitored throughout the whole process. At the end of the experiment, spleen and thymus indices of mice were recorded. Data are showed as the means ± S.E.M. (n = 10). ^#^ *P* < 0.05 and ^##^ *P* < 0.01 versus CTRL group, *P < 0.05 and **P < 0.01 versus model group.

### CSEI promoted peripheral blood cells quantities and the production of murine bone marrow cells

After the last administration, the peripheral blood of mice was collected and analyzed. The quantities of peripheral blood WBC, RBC, HGB, HCT, MCH, and MCHC in the model group were 1.0 × 10^9^/L, 9.3 × 10^12^/L, 135.7 g/L, 40.6%, 14.5 pg, and 334.0 g/L, respectively, which were significantly lower than those of the control group (*P* < 0.05; Table 2). Three weeks of administration of CSEI at doses of 2.25, 4.50, and 9.00 mg/kg strongly reversed this decrease to varying degrees (*P* < 0.05; Table 2), indicating its protective effect on the production of peripheral blood cells in mice with CTX-induced hematopoietic injury.

**Table 2:** Effects of CSEI on peripheral blood cells of CTX-injected mice with hematopoietic injury.

	CTRL	Model	Tα1 (0.24 mg/kg)	CSEI (mg/kg)		
				2.25	4.50	9.00
WBC (×10^9^/L)	3.3 ± 0.3	1.0 ± 0.1^##^	4.7 ± 1.1^*^	3.2 ± 0.3^**^	3.7 ± 0.4^**^	5.0 ± 0.4^**^
RBC (×10^12^/L)	10.4 ± 0.1	9.3 ± 0.1^##^	9.5 ± 0.5	10.1 ± 0.2^*^	9.8 ± 0.2	9.6 ± 0.3
HGB (g/L)	157.0 ± 2.5	135.7 ± 1.2^##^	140.7 ± 4.5	145.7 ± 3.8	149.7 ± 4.3^*^	151.3 ± 2.3^**^
HCT (%)	44.6 ± 0.4	40.6 ± 0.5^##^	41.6 ± 2.0	42.4 ± 1.4	43.4 ± 0.5^*^	42.1 ± 1.0
MCV (fL)	43.1 ± 0.0	43.5 ± 0.2	43.7 ± 0.6	42.4 ± 0.7	44.2 ± 0.2	43.8 ± 0.3
MCH (pg)	15.2 ± 0.1	14.5 ± 0.1^#^	14.8 ± 0.4	14.6 ± 0.4	15.6 ± 0.2^**^	15.7 ± 0.3^*^
MCHC (g/L)	352.0 ± 3.2	334.0 ± 1.5^##^	339.0 ± 5.5	344.0 ± 4.7	346.0 ± 4.0*	359.7 ± 4.3^**^
PLT (×10^9^/L)	1015.0 ± 95.5	1126.3 ± 91.5	1200.5 ± 55.1	1169.7 ± 96.6	1016.3 ± 94.6	1110.7 ± 104.5

100 mg/kg of CTX was intraperitoneally injected to mice except controls for three days. Then Tα1 and CSEI were treated for another 21 days. At the end of the experiment, peripheral blood of mice were collected and analyzed via a blood cell analyzer. Data are showed as the means ± S.E.M. (n = 10). ^#^ *P* < 0.05 and ^##^ *P* < 0.01 versus CTRL group, *P < 0.05 and **P < 0.01 versus model group.

CD45 is a leukocyte common antigen expressed on all leukocytes. Two kinds of leukocytes, i.e., neutrophils and B lymphocytes, in the mice bone marrow were investigated by gating of CD45.

**Figure 3:**
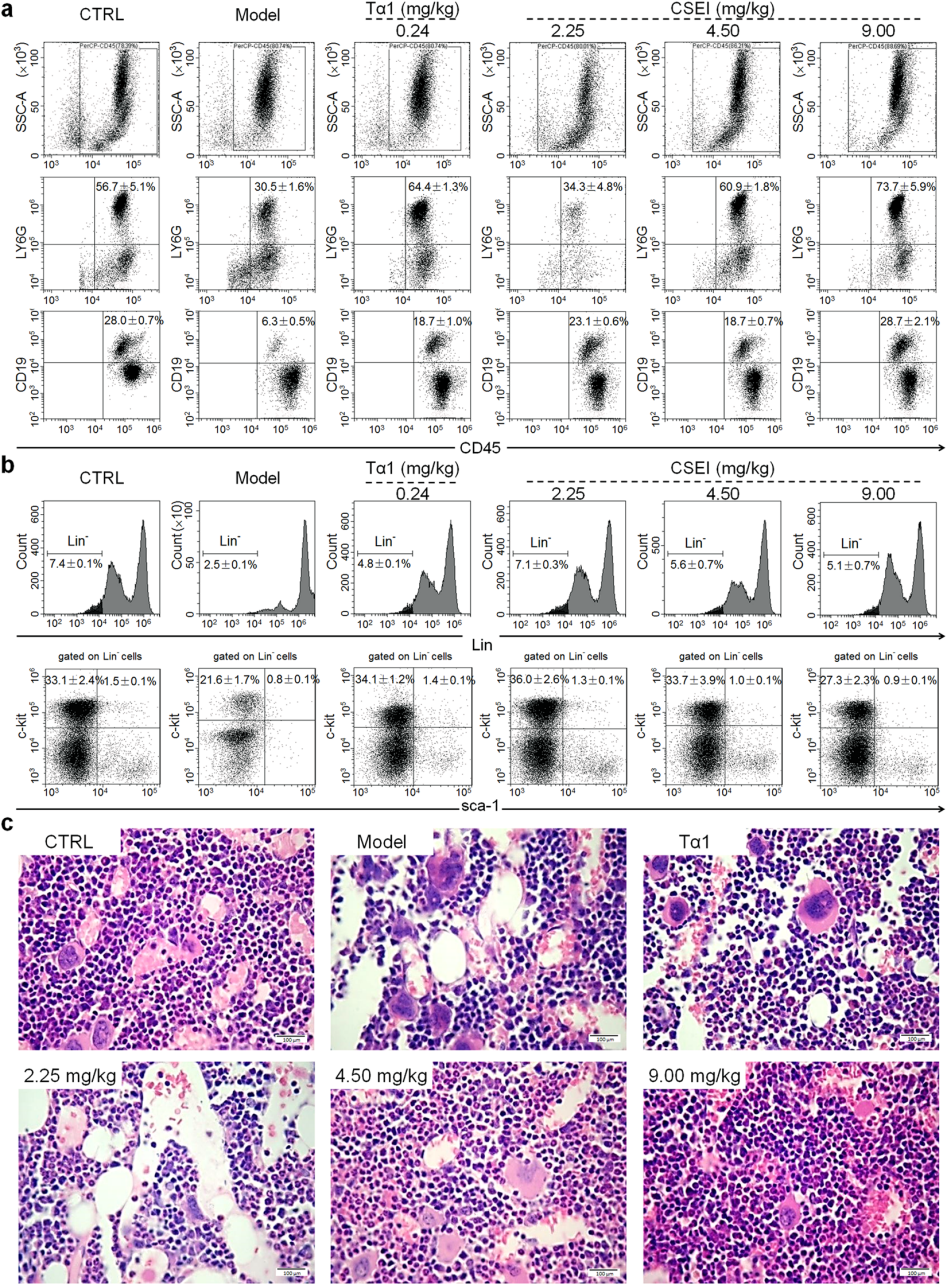
CSEI (2.25, 4.50 and 9.00 mg/kg) enhanced the CTX (100 mg/kg)-induced decrement of leukocyte, HSC/HPC and total bone marrow cellularity production in murine bone marrow of mice after 3-week administration. (**a**) Flow cytometry analysis of the proportion of leukocytes in murine bone marrow. CD45 was used for the sorting of leukocytes. CD45^+^Ly6G^+^ represents neutrophils and CD45^+^CD19^+^ represents B cells. (**b**) The percentage of HSCs (Lin^−^c-kit^+^sca-1^+^) and HPCs (Lin^−^c-kit^+^sca-1^−^) in murine bone marrow of CTX-injected mice were analyzed by flow cytometry assay. (**c**) H&E staning of femurs were observed under a light-microscope digital camera (40×, scale bar: 100 μm) to evaluate the total bone marrow cellularity of bone marrow. The data are shown as the means ± S.E.M. (n = 10).

The expressions of CD45^+^Ly6G^+^ and CD45^+^CD19^+^, represent neutrophils and B lymphocytes respectively, were 56.7% and 28.0% in the control cells respectively, while the corresponding respective percentages in the model group were 30.5% and 6.3% (*P* < 0.01; Fig. 3a and Supplementary Fig. S2). Both 3-week treatment with CSEI and Tα1 attenuated this reduction in bone marrow leukocyte production. CSEI strongly enhanced the production of these two leukocytes in mice with CTX-induced hematopoietic injury (*P* < 0.05; Fig. 3a). The related quantification data is shown in Supplementary Fig. S2.

Hematopoietic stem/progenitor cells (HSC/HPCs) have the potential to differentiate into different mature blood cell lineages^22^. To measure the effect of CSEI on HSPCs, we analyzed the HSCs (Lin^−^c-kit^+^sca-1^+^) and HPCs (Lin^−^c-kit^+^sca-1^−^) in mouse bone marrow. The expression of HSCs and HPCs in control group Lin^−^ bone marrow cells were 1.5% and 33.1%, whereas the corresponding respective percentages in the model group were 0.8% and 21.6% (Fig. 3b and Supplementary Fig. S4). Both CSEI and Tα1 attenuated this reduction in HSCs and HPCs (Fig. 3b and Supplementary Fig. S4). A dose of 2.25 mg/kg CSEI showed the optimum effect and enhanced the production of HSCs in total BMNCs compared to the model group, indicating that CSEI can restore CTX-induced reduction of HSC/HPCs. The absolute numbers of HSCs and HPCs per mouse are shown in Supplementary Fig. S4.

Bone marrow cellularity was analyzed by H&E staining to explore the hematopoietic recovery function of CSEI. In the control mice, the color of the bone marrow tissue was homogeneous, the structures of the periosteum and cavitas medullaris were clear, and the bone marrow cells were identical in distribution (Fig. 3c). In contrast, many vacuolar structures and a low cell density were noted in the bone marrow sections of the model group (Fig. 3c). Compared to the model mice, CSEI showed a dose-dependent increase in bone marrow cell density and a decrease in vacuolization degradation (Fig. 3c).

### CSEI influenced the levels of cytokines in the spleens of the mice

**Figure 4:**
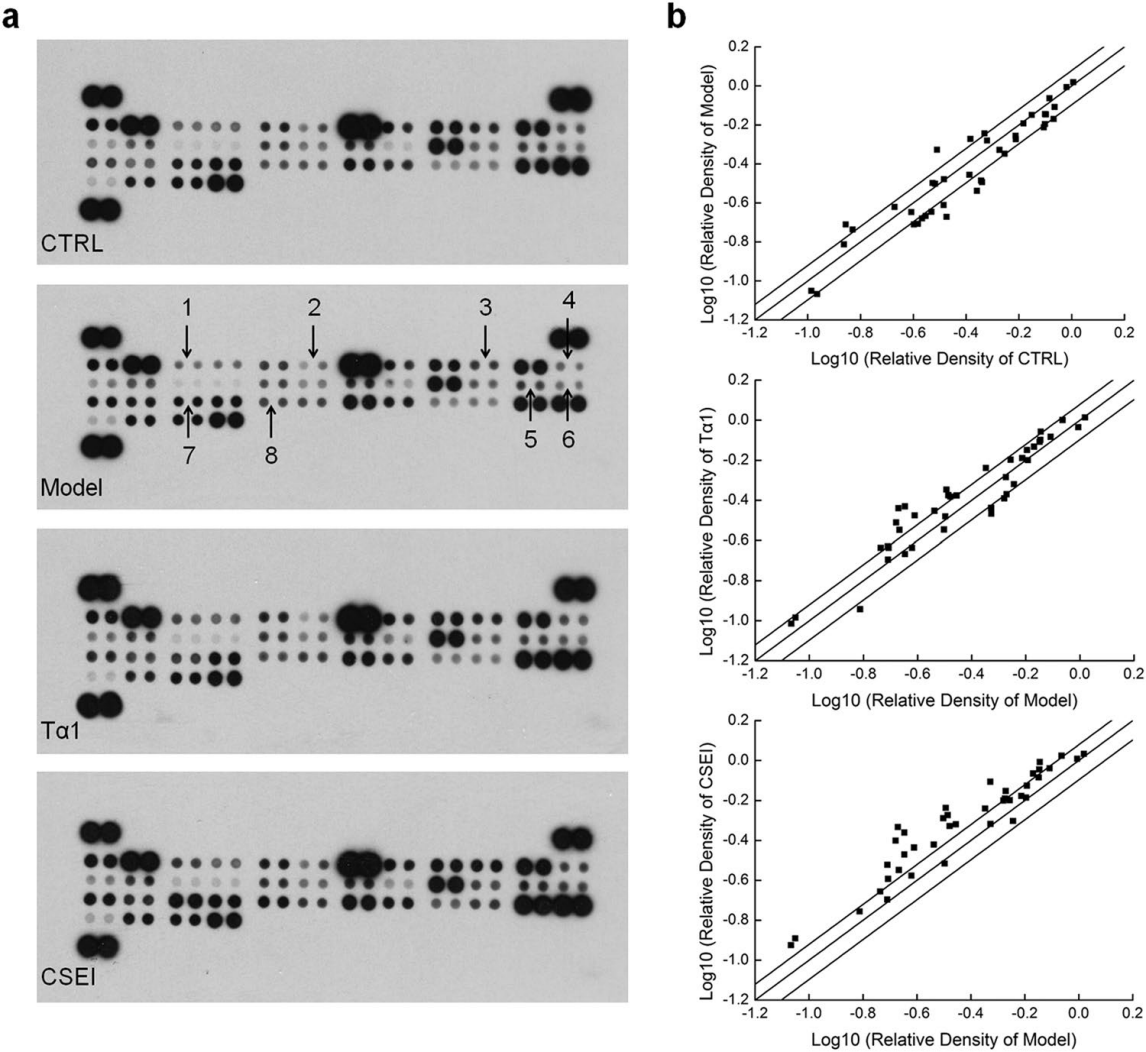
The effects of CSEI (4.50 mg/kg) and Tα1 (0.24 mg/kg) on 40 cytokines in mice spleens detected by Mouse Cytokine Array Panel A Kit. (**a**) Graphical representation of cytokine expressions. The arrows indicate the factors with a change of >50% (CSEI group versus model group). 1. G-CSF; 2. IL-1β; 3. IL-2; 4. IL-23; 5. IL-27; 6. Eotaxin; 7. KC; 8. JE. (**b**) Scatter diagram of 40 cytokines. The relative density is the ratio of the absolute value and the reference spot value.

To systematically investigate the hematopoietic-promoting effects of CSEI and its underlying mechanism, a Mouse Cytokine Array Panel A Kit was used to detect the cytokines in the spleens of the mice. The group receiving CSEI at a dose of 4.50 mg/kg enhanced the levels of 23 hematopoietic cytokines and reduced levels of MIP-1α (Supplementary Table S1). Compared with the model group, CSEI enhanced the hematopoietic cytokines interleukins IL-1β, IL-2, IL-23, IL-27, G-CSF, eotaxin, KC (IL-8) and monocyte chemotactic protein-1 (JE) (Fig. 4; Supplementary Table S1).

### CSEI regulated the levels of hematopoietic cytokines in the plasma

**Figure 5:**
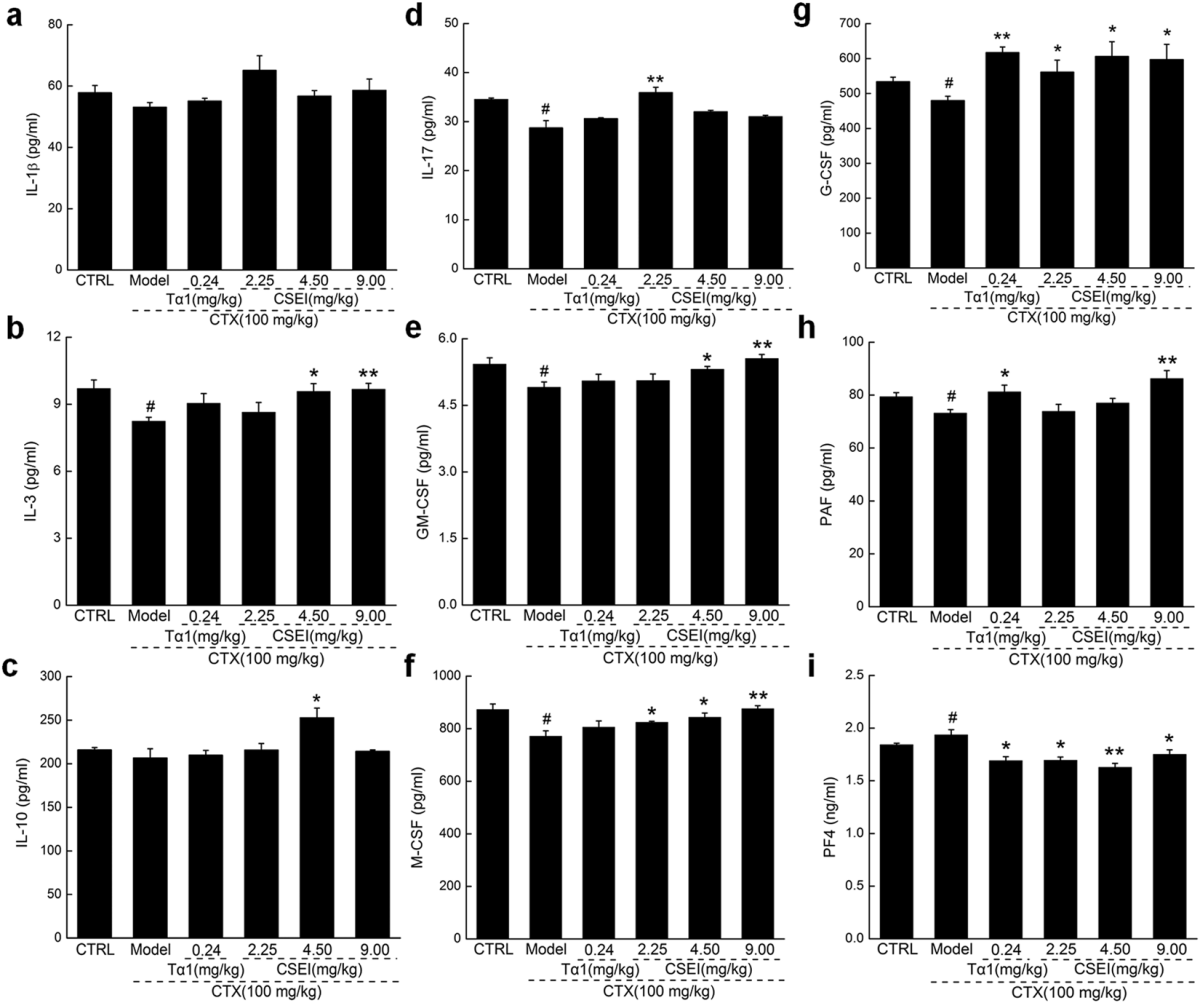
After a 3-day CTX (100 mg/kg) injection and a 21-day CSEI (2.25, 4.5 and 9 mg/kg) treatment, the levels of nine factors related to hematopoietic function including IL-1β (**a**), IL-3 (**b**), IL-10 (**c**), IL-17 (**d**), GM-CSF (**e**), M-CSF (**f**), G-CSF (**g**), PAF (**h**) and PF4 (**i**), in the mice plasma were detected by ELISA method. The data are expressed as the means ± S.E.M. (n = 10). ^#^ *P* < 0.05 versus the control group, *P < 0.05 and **P < 0.01 versus the model group.

Based on the results of proteome profiling, the plasma levels of hematopoietic cytokines including interleukins (IL-1β, IL-3, IL-10, IL-17), colony-stimulating factors (granulocyte colony-stimulating factor: G-CSF, macrophage colony-stimulating factor: M-CSF, granulocyte–macrophage colony-stimulating factor: GM-CSF), platelet activating factor (PAF) and platelet factor 4 (PF4) were analyzed. CTX suppressed the levels of interleukins, especially IL-3 and IL-17, which were strongly enhanced by CSEI to 17.0% (*P* < 0.01; Fig. 5b) and 24.8% (*P* < 0.01; Fig. 5d), respectively.

CSFs, also known as hematopoietic growth factors, promote the differentiation of stem cells and regulate the production of blood cells^23^. Compared with the CTX-treated mice, CSEI increased the plasma levels of GM-CSF, M-CSF, and G-CSF up to 13.2% (*P* < 0.01, Figs. 5e), 13.6% (*P* < 0.01, Fig. 5f), and 26.4% (*P* < 0.05, Fig. 5g), respectively, which were all suppressed after CTX injection (*P* < 0.05, Fig. 5e–g). In addition, CSEI improved the expression of the hematopoietic-positive chemokine PAF up to 17.9% (*P* < 0.01; Fig. 5h) and strongly suppressed the level of the hematopoietic-negative chemokine PF4 up to 15.9% (*P* < 0.01; Fig. 5i) in the CTX mice.

### CSEI regulated the G-CSF-mediated STAT3 signaling pathway

**Figure 6:**
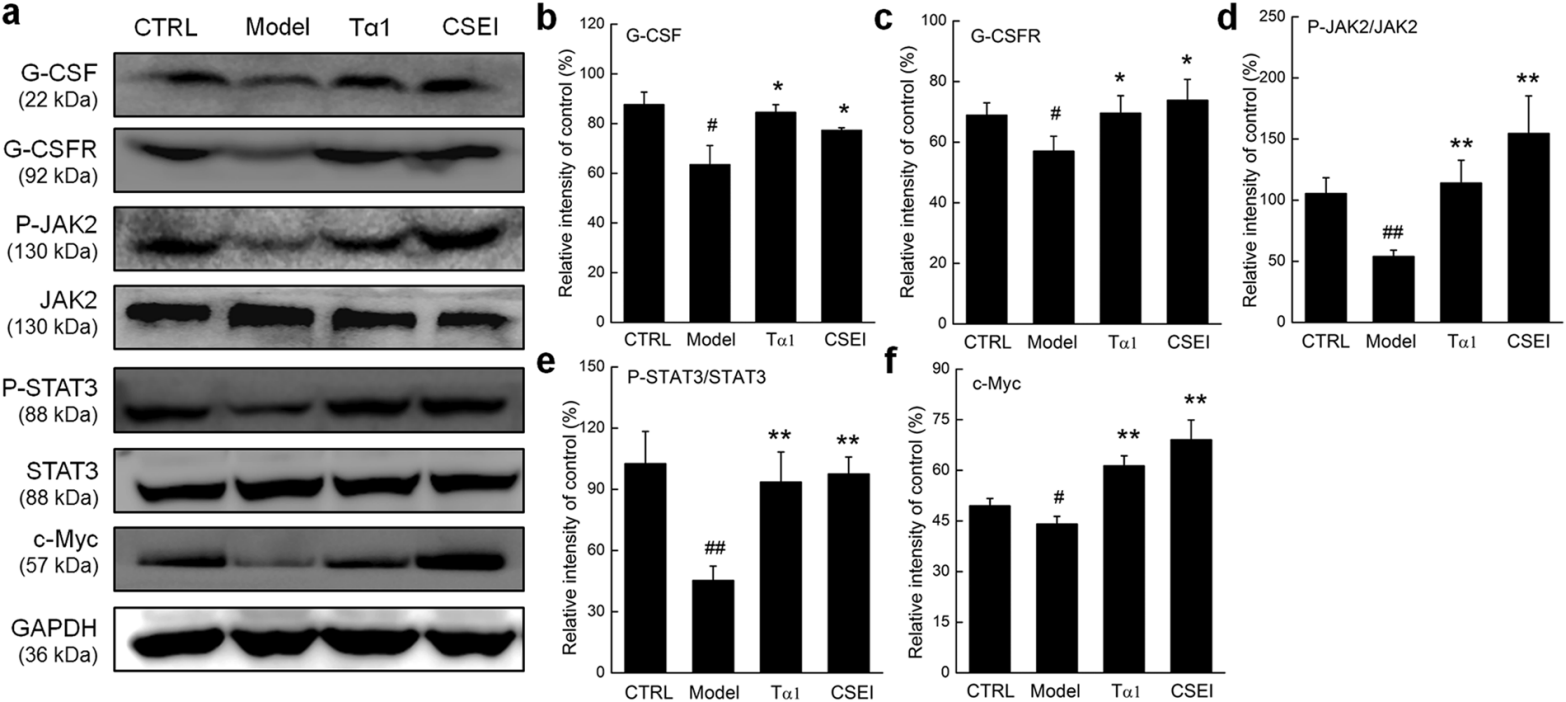
G-CSF-mediated JAK2/STAT3 signaling pathway is, at least, partially involved in the protective effect of CSEI on mice with CTX-induced hematopoietic injury. (**a**) CSEI at a dose of 4.5 mg/kg enhanced the levels of G-CSF, G-CSFR, P-JAK2, P-STAT3 and c-Myc in spleen of CTX-injected mice after a 21-day treatment. The quantitative data of the G-CSF (**b**), G-CSFR (**c**), P-JAK2 (**d**) P-STAT3 (**e**) and c-Myc (**f**) levels were normalized by the corresponding GAPDH and related total protein expressions, and expressed as the percentage of the corresponding relative intensity of the control. Full-length blots are presented in Supplementary Fig. S6a. The data are shown as the means ± S.E.M. (n = 10). ^#^ *P* < 0.05 and ^##^ *P* < 0.01 versus the control group, *P < 0.05 and **P < 0.01 versus the model group.

The expressions of proteins related to G-CSF, granulocyte colony-stimulating factor receptor (G-CSFR) and STAT3 signaling in the spleens of the mice were detected to investigate the mechanisms by which CSEI promoted hematopoiesis. CSEI and Tα1 both enhanced the levels of G-CSF and G-CSFR (*P* < 0.05, Fig. 6a–c). Compared with the CTX-treated mice, CSEI enhanced the phosphorylation of JAK2 and STAT3 (*P* < 0.01, Fig. 6a,d and e). In addition, the levels of c-Myc, the target protein of STAT3, were investigated. CSEI up-regulated the c-Myc level by 56.7% compared with the model group (*P* < 0.01, Fig. 6a and f).

**Figure 7:**
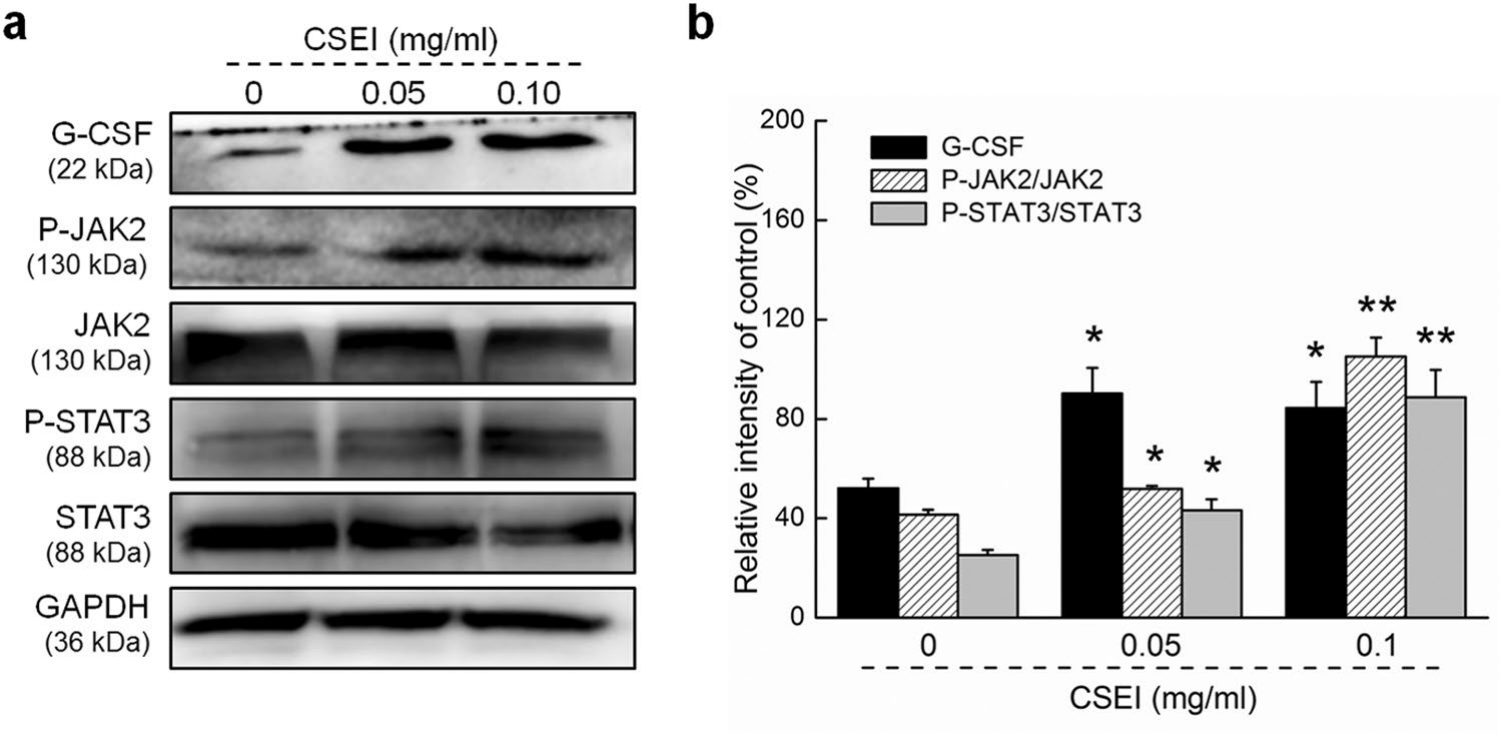
G-CSF-mediated JAK2/STAT3 signaling is promoted by CSEI in the primary cultured BMNCs. The BMNCs were seeded and co-cultured with CSEI (0, 0.05, 0.1 mg/ml) for 24 h. (**a**) The protein expression levels of G-CSF, JAK2, P-JAK2, STAT3, and P-STAT3 were analyzed by western blotting. (**b**) The protein levels were normalized by the corresponding GAPDH and related total protein expressions, and expressed as the percentage of the corresponding relative intensity of the control. Full-length blots are presented in Supplementary Fig. S6b. The data are shown as the means ± S.E.M. (n = 10). *P < 0.05 and **P < 0.01 versus 0 mg/ml CSEI-treated cells.

Similar detection was carried out in primary cultured BMNCs, and the expression levels of G-CSF, P-JAK2, and P-STAT3 were detected after 24 h of CSEI incubation by western blotting. CSEI at 0.1 mg/ml significantly increased the G-CSF, P-JAK2, and P-STAT3 levels by 62.0%, 154.6%, and 251.9%, respectively, compared to the cells that were not treated with CSEI (*P* < 0.05, Fig. 7a and b). CSEI-mediated hematopoiesis promotion may be related to its modulation of G-CSF–mediated STAT3 signaling.

## Discussion

Although chemotherapy is widely used to treat malignancies, the suppression of blood cells and hematopoietic tissue seriously limits its effectiveness and application^24^. CTX, a commonly used alkylating agent in the adjuvant therapy of malignant neoplasms, induces numerous adverse effects, especially immunosuppression and myelosuppression^25, 26^. Via intraperitoneal injection with CTX, a myelosuppressive mouse model was successfully developed through the destruction of bone marrow stroma and microcirculation^27^. We confirmed the protective function of CSEI in hematopoietic cells and in mice with CTX-induced hematopoietic suppression, and found that G-CSF-mediated JAK2/STAT3 signaling was related to this effect.

Within the hematopoietic system, the differentiation of hematopoietic progenitor cells into erythroid and megakaryocytic lineages as well as the production of platelets by mature megakaryocytes play crucial roles in hematopoietic function^28, 29^. RSK1p90 and intranuclear transcription factor ELK1, recognized as downstream proteins of the MAPK pathway, are involved in cell proliferation and migration^30, 31^. Protooncogene c-Myc, a member of the Myc family, is critical for maintaining correct hematopoiesis, helping to regulate differentiation, proliferation and the balance of self-renewal of stem cells. The down-regulation of Myc in leukemia and lymphoma suppresses the proliferation of hematopoietic cells, and even inhibts their terminal differentiation^32^. Additionally, vertebrate transcription factors GATA-1 and GATA-2 are essential to the differentiation of erythroid lineages and maturation of megakaryocytes through the regulation of genes^33, 34^. Saponins from *Panax notoginseng* promote the proliferation and differentiation of hematopoietic cells via increasing the expression of GATA-1 and GATA-2^35^. Combined with our present data, CSEI has been verified to promote the proliferation of CHRF and K562 cells and the erythroid differentiation of K562 cells. All of these results indicate that CSEI exerts potential protective effects on the hematopoietic system.

Disorders in the bone marrow hematopoietic systemare often accompanied by extramedullary hematopoiesis (EMH) in the liver, spleen or spine of patients^36^. CTX induces EMH in the spleens of mice, followed by spleen enlargement^37^. Encouragingly, CSEI suppressed the CTX-induced enlargement of the mouse spleens, implying that it improves the hematopoietic system and reverses the symptoms of EMH. Multipotent hematopoietic stem cells in bone marrow have the capacity for self-renewal and differentiation. Within the bone marrow microenvironment, hematopoietic stem cells can be differentiated into directional progenitor cells and precursor cells, and finally differentiated or matured into various hemocytes with different functions^38^, including neutrophils and B cells. Studies have shown that lentinan can increase the bone marrow neutrophils in mice with therarubicin-induced myelosuppression^39, 40^. The antioxidant chlorophyllin enhances bone marrow granulopoiesis, substantially increases peripheral blood neutrophils and further alleviates radiation-induced hematopoietic syndrome^41^. In this study, CSEI reversed the CTX-induced reduction of peripheral blood cells, HSPCs, neutrophils and B cells in bone marrow. Bone marrow cellularity was also improved after CSEI treatment. In summary, CSEI ameliorated CTX-induced myelosuppression in mice.

Among 40 kinds of cytokines and chemokines, we found that CSEI markedly regulated 8 of them, including interleukins and colony-stimulating factors, in the spleens of mice. By a feedback loop, HSPCs express a range of hematopoietic cytokine receptors that control the differentiation of HSPCs into mature hemocytes^42, 43^. Among the factors found to be regulated, PF4 reduced the production of platelets through the inhibition of megakaryocyte progenitors *in vivo* ^44^. PAF, a phospholipid mediator that plays vital roles in mature leukocytes, acts directly on hematopoiesis by influencing the growth of hematopoietic progenitor cells, and indirectly through regulating the release of cytokine in bone marrow stromal cells^45^. Eotaxin can induce eosinophil production, and Lambert *et al*. observed that mouse KC (IL-8) played a significant role in the process of CpG-ODNs mobilizing HPCs into peripheral blood^46^, while monocyte chemotactic protein-1 (MCP-1/JE) influenced the proliferation and clonal expansion of murine HPCs^47^. Interleukins and colony-stimulating factors are two major types of hematopoietic growth factors controlling hematopoiesis through a network^48^. IL-lβ, responsible for the cytokine cascade process, stimulates the production of IL-17 and further activates the release of G-CSF, or directly augments the production of G-CSF^49, 50^. IL-3 is a multipotent hematopoietic growth factor that stimulates the proliferation of stem cells; however, it depends on other cytokines such as G-CSF and IL-5 when promoting hematopoiesis^51^. IL-10 can up-regulate the levels of G-CSF and M-CSF by inhibiting the synthesis of TGF-β^52^. As reported, IL-17 regulates hematopoiesis mainly through stimulating fibroblasts to secrete secondary hematopoietic cytokines including G-CSF and IL-6^53^. The IL-17/G-CSF–cytokine-controlled loop can regulate neutrophil homeostasis^54^. Our present data suggest that G-CSF may be an indispensable factor in CSEI-mediated protection in mice with injured hematopoietic functions.

As reported previously, G-CSF specifically binds to its plasmalemma receptor, G-CSFR, to induce dimerization, and further causes the activation of intracellular STATs, especially STAT3, which is responsible for cell proliferation and differentiation^55^. In this study, CSEI stimulated the phosphorylation of STAT3 and its upstream extracellular protein JAK2 in the spleens of CTX-injected mice with hematopoietic suppression and in the primary cultured BMNCs. The activated STAT3 translocated into the nucleus, binding to the target DNA-binding elements, especially c-Myc. As the product of a transcription factor, c-Myc is involved in the regulation of self-renewal and differentiation of hematopoietic stem cells^32, 56^.

In conclusion, we successfully confirmed the protective effect of CSEI on hematopoiesis *in vitro* and *in vivo*. All of the data suggest that this effect is related to the G-CSF-mediated JAK2/STAT3 pathway.

## Materials and Methods

### Cell culture

CHRF (CRL10107) and K562 (CCL-243™)cell lines, obtained from the American Type Culture Collection (ATCC; USA), were cultured in RPMI 1640 medium plus 10% fetal bovine serum (FBS), 100 U/ml penicillin and 100 μg/ml streptomycin at 37 °C in a fully humidified incubator under 5% CO_2_ and 95% air.

The bone marrow cells were flushed from the bones of healthy BALB/c mice with Iscove’s Modified Dulbecco’s Medium using a 1-ml syringe with a 21-gauge needle. BMNCs were collected with red blood cell lysis buffer (Gibco BRL, Grand Island, NY) according to the manufacturer’s protocol. The BMNCs were then cultured at a density of 2 × 10^6^ cells/well in a 6-well plate in IMDM plus 20% FBS, 100 U/ml penicillin, and 100 μg/ml streptomycin at 37 °C in a fully humidified incubator under 5% CO_2_ and 95% air.

Cell culture reagents were obtained from Gibco BRL (Grand Island, NY).

### XTT assay

The cell proliferation capacity of K562 and CHRF cells was detected by XTT assay according to a previously described method^57^. Briefly, CHRF and K562 cells were seeded into 96-well plates at 1 × 10^5^ cells/ml and incubated with 0, 0.05 and 0.1 mg/ml of CSEI (supplied by Jilin Aodong Medicine Industry Co., Ltd., Jilin, China) for 24 h. Then, 50 μl of 37 °C pre-warmed reactant containing 50 μg of sodium 3′-[1-[(phenylamino)-carbonyl]-3,4-tetrazolium]-bis (4-methoxy-6-nitro) benzene-sulfonic acid hydrate (XTT) and 0.38 μg of phenazine methosulfate (PMS) which were obtained from Sigma-Aldrich was added. After a 4-h incubation period, the absorbance was measured by a Synergy^TM4^ Microplate Reader (BioTek Instruments, Winooski, VT) at a wavelength of 450 nm.

### Erythroid differentiation of K562 cells

Benzidine staining is commonly used to identify hemoglobin-containing cells, and the assay was performed according to the previous method with slight modification^58^. Briefly, CHRF and K562 cells were seeded into six well plates at a density of 2 × 10^5^ cells/well. The cells were incubated with an equal volume of CSEI/1640 medium or 1640 medium for 24 h. After three washes with phosphate-buffered saline (PBS), the cells were suspended to 1 × 10^5^ cells/500 μl and stained with 14 μl of 0.4% benzidine and 1 μl of 30% hydrogen peroxide for 2 min, followed by adding l μl of 5% sodium nitroprusside. After a 15-min reaction period, the cells were examined using a light-microscope digital camera (Nikon Instruments, Tokyo, Japan). Benzidine-positive cells showed dark blue, while benzidine-negative cells showed light yellow. Groups of 200 cells were counted and the proportion of positive cells in each group was calculated.

Glycophorin A (CD235a) was used to analyze the proportion of erythrocytes in K562 cell via flow cytometry. K562 cells were seeded into 96-well plates at 1 × 10^5^ cells/ml and incubated with 0, 0.05, and 0.1 mg/ml CSEI for 24 h. K562 cell suspensions (100 μl) containing 1 × 10^6^ cells were stained with PE-conjugated anti-human CD235a (349106, San Diego, CA) antibody. PE-conjugated anti-mouse IgG2a (400213) was set as the isotype control. Analysis was performed on a CytoFLEX flow cytometer (Beckman Coulter) according to the manufacturer’s instructions.

### Animals and experimental design

The Institution Animal Ethics Committee of Jilin University approved all of the *in vivo* studies described and all methods were performed in accordance with the relevant guidelines. BALB/c mice (4–6 weeks; 18–22 g) purchased from Norman Bethune University of Medical Science, Jilin University, China (SCXK(JI)-2016-003) were brought naturally to ambient environmental conditions at 23 ± 1 °C with a 12-h photoperiod, and fed autoclaved standard chow and water *ad libitum*.

120 mice (equal numbers of males and females) were randomly divided into six groups (n = 20). The control mice were intraperitoneally injected with 10 ml/kg of normal saline throughout the experiment. 100 mg/kg of CTX (Sigma-Aldrich, USA) was intraperitoneally injected into all of the mice except those in the control group for 3 days. The positive group was injected with thymosin alpha 1 (Tα1, purchased from Harbin Pharmaceutical Group Biological Engineering Co., Ltd.) at 0.24 mg/kg subcutaneously twice a week, and the CSEI-treated groups were intraperitoneally injected with CSEI at doses of 2.25, 4.50 and 9.00 mg/kg once a day for 3 weeks. To prevent recovery of hematopoietic function, all mice except the control group were intraperitoneally injected with 100 mg/kg of CTX at the 15^th^ day.

The bodyweights of the mice were recorded at the 1^st^, 4^th^, 11^th^, 18^th^ and 25^th^ days. After the last administration, the mice were euthanized, and the spleen and thymus tissues were collected and weighed immediately. The organ index was calculated by the following formula: organ index (%) = organ weight/body weight × 100%.

### Peripheral blood cells analysis

Peripheral blood from the orbital venous plexus of the mice was collected into clean tubes with ethylenediaminetetraacetic acid (EDTA) after the last administration. Blood samples (300-μl) were analyzed with a HEMAVET 950 fully automatic blood cell analyzer (Drew Scientific Group, Dallas, TX).

### Isolation of murine bone marrow and flow cytometry analysis

The femurs and tibia of the mice were sterilely obtained immediately after the mice were euthanized. The bone marrow cells were flushed from the bones by Iscove’s Modified Dulbecco’s Medium (IMDM) using a 1-ml syringe with a 21-gauge needle. Then BMNCs were prepared with mouse lymphocyte separation medium according to the manufacturer’s protocol. And cells were fixed with 4% paraformaldehyde at 4 °C.

Then, 100 μl BMNCs suspensions containing 1 × 10^6^ cells were stained with PerCP-conjugated anti-mouse CD45 (103129), PE-conjugated anti-mouse Ly-6G (127607), FITC-conjugated anti-mouse/human CD11b (101205), APC-conjugated anti-mouse CD19 (152410), FITC-conjugated anti-mouse Lineage Cocktail (133302), PE-conjuated anti-mouse Ly-6A/E (sca-1) (108108) or APC-conjugated anti-mouse CD117 (c-kit) (135108) antibodies, respectively. PerCP-conjugated anti-rat IgG2b (400629), PE-conjugated anti-rat IgG2a (400507), APC-conjugated anti-rat IgG2a (400511), APC-conjugated anti-rat IgG2b (400611), FITC-conjugated anti-rat IgG2b (400605), PE-conjugated anti-mouse IgG2a (400213) and FITC-conjugated anti-rat IgG2a (400505) were set as isotype controls. Analysis was performed on a CytoFLEX flow cytometer according to the manufacturer’s instructions. The antibodies were obtained from Biolegend (San Diego, CA).

### Histological examination of bone marrow

Mouse femurs were obtained immediately after euthanasia and fixed in 4% paraformaldehyde. After incubation with decalcification solution for 7 days, the tissues were embedded in paraffin, sliced to a thickness of 5 μm, and stained with H&E. The histologic sections were examined under a light-microscope digital camera (Nikon Instruments, Tokyo, Japan).

### Proteome profiling of spleens

Forty different cytokines of the mice were analyzed using a Mouse Cytokine Array Panel A Kit (R&D Systems, Minneapolis, MN). Briefly, the spleen was excised and homogenized in RIPA lysis buffer (Sigma-Aldrich, USA) containing1% protease inhibitor cocktail (Sigma-Aldrich, USA) and 2% phenylmethanesulfonyl fluoride (Sigma-Aldrich, USA). The homogenate was centrifuged at 10,000 rpm for 5 min, and the protein concentration of the supernatant was quantitated by a BCA protein assay kit (Merck Millipore, Billerica, MA). Membranes containing 40 different cytokine antibodies were blocked with BSA for 1 h at room temperature and then incubated with 100 μg of protein supernatant mixed with a cocktail of biotinylated detection antibodies. Streptavidin–HRP and chemiluminescence were used to detect the antibodies bound to the membrane antibodies. The membranes were then exposed and quantified using Image J software (National Institutes of Health, Bethesda, MD).

### Plasma cytokine detection

At the end of the experiment, blood was sampled from the orbital venous plexus and centrifuged at 3,000 rpm for 10 min to obtain the plasma after standing at room temperature for 30 min. Enzyme-linked immunosorbent assay (ELISA) kits were applied to determine the levels of plasma cytokines including G-CSF (MCS00), M-CSF (MMC00), GM-CSF (MGM00), PF4 (MCX400) and the interleukins IL-1β (MLB00C), IL-3 (M3000), IL-10 (M1000B) and IL-17 (M1700), which were obtained from R&D Systems (Minneapolis, MN), and PAF (abx254319), which was obtained from Abbexa (Cambridge, UK). All of the procedures were performed according to the kit manufacturer’s protocol.

### Western blotting

K562 and CHRF cells were seeded at a concentration of 6 × 10^5^ cells/well, and BMNCs were seeded at a concentration of 2 × 10^6^ cells/well into 6-well plates for 24 h. The cells and spleen tissues were collected and lysed in RIPA lysis buffer containing 1% protease inhibitor cocktail (Sigma-Aldrich, USA) and 2% phenylmethanesulfonyl fluoride (Sigma-Aldrich, USA). The lysates were centrifuged, and the total protein concentration of the supernatant was quantitated by a BCA protein assay kit. 40 μg of protein was separated by 10% SDS-PAGE and transferred onto a PVDF membrane (0.45 μm, Merck Millipore, Billerica, MA). After a 2-h blocking period with BSA at room temperature, the membrane was incubated with the following primary antibodies at 4 °C overnight: RSK1p90 (ab32526), phosphor(P)-RSK1p90 (ab32413), ELK1 (ab188316), c-Myc (ab32072), GATA-1 (ab89505), GATA-2 (ab109241), G-CSF (ab181053), G-CSFR (ab19479), JAK2 (ab108596), phosphor (P)-JAK2 (ab32101), STAT3 (ab119352), phosphor (P)-STAT3 (ab76315) and GAPDH (ab181602), all obtained from Abcam (Cambridge, MA). Horseradish peroxidase (HRP)-conjugated secondary antibodies were then used to bind the primary antibodies at 4 °C for 4 h. The corresponding protein expressions were measured by an ECL detection kit (Merck Millipore, Billerica, MA) and visualized by an imaging system (BioSpectrum600). The pixel density was quantified using Image J software (National Institutes of Health, Bethesda, MD).

### Statistical analysis

One-way analysis of variance (ANOVA) was conducted to determine statistical significance, followed by post-hoc multiple comparisons (Dunn’s test) using SPSS 16.0 software (IBM Corporation, Armonk, NY). The value of *P* < 0.05 was considered significant.

### Data Availability

All data generated or analysed during this study are included in this published article and its Supplementary Information files.

### Ethical Approval

The Animal Ethics Committee of Jilin University approved the experimental animal protocol.

## Electronic supplementary material

Supplementary Information

## References

[1] Vasil’eva TV, Tskhovrebova AZ, Michurina TV, Khrushchov NG (1977). [Immunofluorescent study of the hemopoietic organs of xenogeneic mouse radiation chimeras]. Ontogenez.

[2] Fauser AA, Messner HA (1979). Identification of megakaryocytes, macrophages, and eosinophils in colonies of human bone marrow containing neurtophilic granulocytes and erythroblasts. Blood.

[3] Faustman DL (2005). Regenerative medicine: Stem cell research turns to the spleen. Discovery medicine.

[4] Sorby R, Wien TN, Husby G, Espenes A, Landsverk T (2005). Filter function and immune complex trapping in splenic ellipsoids. Journal of comparative pathology.

[5] O’Malley DP (2007). Benign extramedullary myeloid proliferations. Modern pathology: an official journal of the United States and Canadian Academy of Pathology, Inc.

[6] Cluitmans FH (1997). The role of cytokines and hematopoietic growth factors in the autocrine/paracrine regulation of inducible hematopoiesis. Annals of hematology.

[7] Sefc L, Psenak O, Sykora V, Sulc K, Necas E (2003). Response of hematopoiesis to cyclophosphamide follows highly specific patterns in bone marrow and spleen. Journal of hematotherapy & stem cell research.

[8] Shao L (2013). Hematopoietic stem cell senescence and cancer therapy-induced long-term bone marrow injury. Translational cancer research.

[9] Reigle BS, Dienger MJ (2003). Sepsis and treatment-induced immunosuppression in the patient with cancer. Critical care nursing clinics of North America.

[10] Cetean S (2015). The importance of the granulocyte-colony stimulating factor in oncology. Clujul medical (1957).

[11] Zhao CL (2015). Recombinant Human Granulocyte Colony-Stimulating Factor Promotes Preinvasive and Invasive Estrogen Receptor-Positive Tumor Development in MMTV-erbB2 Mice. Journal of breast cancer.

[12] Sun, Y., Wang, J., Li, M. & Zhou, C. Calf spleen extractive injection combined with chemotherapy in the clinical efficacy of the treatment of advanced non-small cell lung cancer. *Chinese Journal of Cancer*, 442–445 (2008).

[13] Liu, J. & Wu, G. Calf spleen extractive injection on immune function in patients with lung cancer. *Journal of Qiqihar Medical School*, 1638–1639 (2015).

[14] Jia D (2016). Investigation on Immunomodulatory Activity of Calf Spleen Extractive Injection in Cyclophosphamide-induced Immunosuppressed Mice and Underlying Mechanisms. Scandinavian journal of immunology.

[15] Jia D (2016). Calf Spleen Extractive Injection (CSEI), a small peptides enriched extraction, induces human hepatocellular carcinoma cell apoptosis via ROS/MAPKs dependent mitochondrial pathway. Journal of Pharmacological Sciences.

[16] Xia, L. & Sun, S. Clinical Observation on Treatment of 30 Cases of Chronic Aplastic Anemia with Calf Spleem Extractive Injection. *Tumor*, 602 (2009).

[17] Chen, J. & Wei, L. Clinical observation of calf spleen extractive injection combined with EPO in the treatment of anemia induced by cancer. *Medical Innovation of China*, 18–19 (2011).

[18] Duncan MT (2014). Dynamic transcription factor activity profiles reveal key regulatory interactions during megakaryocytic and erythroid differentiation. Biotechnology and bioengineering.

[19] Reinheckel, T., Ullrich, O., Sitte, N. & Grune, T. Differential Impairment of 20S and 26S Proteasome Activities in Human Hematopoietic K562 Cells during Oxidative Stress. *Archives of biochemistry and biophysics* **377**, 65–68, doi:10.1006/abbi.2000.1717 (2000). 10.1006/abbi.2000.1717 10775442

[20] Lachmann N (2015). Tightly regulated ‘all-in-one’ lentiviral vectors for protection of human hematopoietic cells from anticancer chemotherapy. Gene therapy.

[21] Kim JY (2010). Molecular mechanisms of cellular proliferation in acute myelogenous leukemia by leptin. Oncology reports.

[22] Lee G-W (2004). *In vivo* efficacy of recombinant leukotactin-1 against cyclophosphamide. Biotechnology and Bioprocess Engineering.

[23] Bath, P. M., Sprigg, N. & England, T. Colony stimulating factors (including erythropoietin, granulocyte colony stimulating factor and analogues) for stroke. *The Cochrane database of systematic reviews*, CD005207, doi:10.1002/14651858.CD005207.pub4 (2013). 10.1002/14651858.CD005207.pub4 PMC11441151 23797623

[24] Liu M (2014). Hematopoietic effects and mechanisms of Fufang ejiao jiang on radiotherapy and chemotherapy-induced myelosuppressed mice. Journal of ethnopharmacology.

[25] Woolley PV, Ayoob MJ, Smith FP, Dritschilo A (1983). Clinical trial of the effect of S-2-(3-aminopropylamino)-ethylphosphorothioic acid (WR-2721) (NSC 296961) on the toxicity of cyclophosphamide. Journal of clinical oncology: official journal of the American Society of Clinical Oncology.

[26] Schein PS, Winokur SH (1975). Immunosuppressive and cytotoxic chemotherapy: long-term complications. Annals of internal medicine.

[27] Xie RF (1985). [Effect of cyclophosphamide on the bone marrow hematopoiesis in the mouse]. Zhonghua zhong liu za zhi [Chinese journal of oncology].

[28] Huang MX, Liu WJ (2009). [Effect of cluster a in Hox gene on proliferation and differentiation of hematopoietic stem/progenitor cells and its relation to leukemia–review]. Zhongguo shi yan xue ye xue za zhi / Zhongguo bing li sheng li xue hui = Journal of experimental hematology / Chinese Association of Pathophysiology.

[29] Pietrzyk-Nivau, A. et al. Three-Dimensional Environment Sustains Hematopoietic Stem Cell Differentiation into Platelet-Producing Megakaryocytes. *PloS one* **10**, doi:10.1371/journal.pone.0136652 (2015). 10.1371/journal.pone.0136652 PMC4552162 26313154

[30] Gogl G (2016). Structural Basis of Ribosomal S6 Kinase 1 (RSK1) Inhibition by S100B Protein: MODULATION OF THE EXTRACELLULAR SIGNAL-REGULATED KINASE (ERK) SIGNALING CASCADE IN A CALCIUM-DEPENDENT WAY. The Journal of biological chemistry.

[31] Donyo M, Hollander D, Abramovitch Z, Naftelberg S, Ast G (2016). Phosphatidylserine enhances IKBKAP transcription by activating the MAPK/ERK signaling pathway. Human molecular genetics.

[32] Delgado MD, Leon J (2010). Myc roles in hematopoiesis and leukemia. Genes & cancer.

[33] Huang Z (2009). GATA-2 reinforces megakaryocyte development in the absence of GATA-1. Molecular and cellular biology.

[34] Bresnick EH, Katsumura KR, Lee HY, Johnson KD, Perkins AS (2012). Master regulatory GATA transcription factors: mechanistic principles and emerging links to hematologic malignancies. Nucleic acids research.

[35] Sun X, Gao RL, Lin XJ, Xu WH, Chen XH (2013). Panax notoginseng saponins induced up-regulation, phosphorylation and binding activity of MEK, ERK, AKT, PI-3K protein kinases and GATA transcription factors in hematopoietic cells. Chinese journal of integrative medicine.

[36] Qiu X (2012). Oestrogen-deficiency inducing haematopoiesis dysfunction via reduction in haematopoietic stem cells and haematopoietic growth factors in rats. International journal of experimental pathology.

[37] Liu HH, Chen FP, Liu RK, Lin CL, Chang KT (2015). Ginsenoside Rg1 improves bone marrow haematopoietic activity via extramedullary haematopoiesis of the spleen. Journal of cellular and molecular medicine.

[38] Frasca D (2000). Hematopoietic reconstitution after lethal irradiation and bone marrow transplantation: effects of different hematopoietic cytokines on the recovery of thymus, spleen and blood cells. Bone marrow transplantation.

[39] Ma RM (2016). Prognostic Value of Chemotherapy-Induced Neutropenia at the First Cycle in Invasive Breast Cancer. Medicine.

[40] Liu Q (2016). Lentinan mitigates therarubicin-induced myelosuppression by activating bone marrow-derived macrophages in an MAPK/NF-kappaB-dependent manner. Oncology reports.

[41] Suryavanshi S (2015). Amelioration of radiation-induced hematopoietic syndrome by an antioxidant chlorophyllin through increased stem cell activity and modulation of hematopoiesis. Free radical biology & medicine.

[42] Hui CC, McNagny KM, Denburg JA, Siracusa MC (2015). *In situ* hematopoiesis: a regulator of TH2 cytokine-mediated immunity and inflammation at mucosal surfaces. Mucosal immunology.

[43] Broxmeyer HE (2008). Chemokines in hematopoiesis. Current opinion in hematology.

[44] Lambert MP (2007). Platelet factor 4 is a negative autocrine *in vivo* regulator of megakaryopoiesis: clinical and therapeutic implications. Blood.

[45] Denizot Y, Guglielmi L, Donnard M, Trimoreau F (2003). Platelet-activating factor and normal or leukaemic haematopoiesis. Leukemia & lymphoma.

[46] Nardini E (2005). CpG-oligodeoxynucleotides induce mobilization of hematopoietic progenitor cells into peripheral blood in association with mouse KC (IL-8) production. Journal of cellular physiology.

[47] Xu YX, Talati BR, Janakiraman N, Chapman RA, Gautam SC (1999). Growth Factors: Production of Monocyte Chemotactic Protein-1 (MCP-1/JE) by Bone Marrow Stromal Cells: Effect on the Migration and Proliferation of Hematopoietic Progenitor Cells. Hematology (Amsterdam, Netherlands).

[48] Sachs, L. & Lotem, J. The network of hematopoietic cytokines. *Proceedings of the Society for Experimental Biology and Medicine*. *Society for Experimental Biology and Medicine (New York, NY)* **206**, 170–175 (1994). 10.3181/00379727-206-43736 8016148

[49] Capitano ML (2012). Elevating body temperature enhances hematopoiesis and neutrophil recovery after total body irradiation in an IL-1-, IL-17-, and G-CSF-dependent manner. Blood.

[50] Kennedy SM, Borch RF (1999). IL-1beta mediates diethyldithiocarbamate-induced granulocyte colony-stimulating factor production and hematopoiesis. Experimental hematology.

[51] Szygula Z (2014). Hematological parameters, and hematopoietic growth factors: EPO and IL-3 in response to whole-body cryostimulation (WBC) in military academy students. PloS one.

[52] Van Vlasselaer, P., Falla, N., Van Den Heuvel, R., Dasch, J. & de Waal Malefijt, R. Interleukin-10 stimulates hematopoiesis in murine osteogenic stroma. *Clinical orthopaedics and related research*, 103–114 (1995). 7641467

[53] Fossiez F (1996). T cell interleukin-17 induces stromal cells to produce proinflammatory and hematopoietic cytokines. The Journal of experimental medicine.

[54] Krstic A, Mojsilovic S, Jovcic G, Bugarski D (2012). The potential of interleukin-17 to mediate hematopoietic response. Immunologic research.

[55] Kusano K (2004). A potential therapeutic role for small nonpeptidyl compounds that mimic human granulocyte colony-stimulating factor. Blood.

[56] Lui GY (2015). Novel thiosemicarbazones regulate the signal transducer and activator of transcription 3 (STAT3) pathway: inhibition of constitutive and interleukin 6-induced activation by iron depletion. Molecular pharmacology.

[57] Scudiero DA (1988). Evaluation of a soluble tetrazolium/formazan assay for cell growth and drug sensitivity in culture using human and other tumor cell lines. Cancer research.

[58] Lam LT, Ronchini C, Norton J, Capobianco AJ, Bresnick EH (2000). Suppression of erythroid but not megakaryocytic differentiation of human K562 erythroleukemic cells by notch-1. The Journal of biological chemistry.

