# Supplementary material for: Calf Spleen Extractive Injection protects mice against cyclophosphamide-induced hematopoietic injury through G-CSF-mediated JAK2/STAT3 signaling
Source: Sci Rep. 2017 Aug 21;7:8402. doi: 10.1038/s41598-017-08970-3 (PMC5566473; doi:10.1038/s41598-017-08970-3)
Supplement: Supplementary file 1 — Supplementary Information [file 41598_2017_8970_MOESM1_ESM.pdf]

Calf Spleen Extractive Injection protects mice against cyclophosphamide-induced hematopoietic injury through G-CSF-mediated JAK2/STAT3 signaling

Wenqian Lu[1], Dongxu Jia[1], Shengshu An[1], Ming Mu[1], Xinan Qiao[1], Yan Liu[1], XinLi[2], Di Wang[1,2]*

1. School of Life Sciences, Jilin University, Changchun, 130012,China;

2. Zhuhai College of Jilin University, Jilin University, Zhuhai, 519000, China

- Corresponding author:

Di Wang, Ph.D
School of Life Sciences, Jilin University
Changchun City, Jilin Province,P.R.China.
Email: jluwangdi@outlook.com

Wenqian Lu, Email: luwq15@mails.jlu.edu.cn

DongxuJia, Email: jiadx14@mails.jlu.edu.cn

ShengshuAn, Email: anss15@mails.jlu.edu.cn

Ming Mu, Email: muming1314@mails.jlu.edu.cn

XinanQiao, Email: qiaoxn1314@mails.jlu.edu.cn

Yan Liu, Email: liuyaorui@126.com

Xin Li, Email: lix15@mails.jlu.edu.cn

Supplementary Data

Materials and Methods

Cell culture. CHRF (CRL10107) and K562 (CCL-243™) cell lines, obtained from the American Type Culture Collection (ATCC; USA), were cultured in RPMI 1640 medium plus 10% fetal bovine serum, 100 U/ml penicillin and 100 μg/ml streptomycin at 37°C in a fully humidified incubator under 5% CO_2 and 95% air. Cell culture reagents were obtained from Gibco BRL (Grand Island, NY).

Apoptosis analysis by Annexin V/PI staining. K562 and CHRF cells were seeded into 96-well plates at 2×10^5 cells/ml and incubated with 0, 0.05 and 0.1 mg/ml CSEI (supplied by Jilin Aodong Medicine Industry Co., Ltd., Jilin, China) for 24 h. Cells were collected and washed with PBS and resuspended in 1% FBS. Then, 100 μl of cells suspension containing 1×10^5 cells were stained with propidium iodide (PI) and/or Annexin V for 20 min at room temperature. Analysis was performed via Muse™ Cell Analyzer from Millipore (Billerica, MA).

Statistical analysis. One-way analysis of variance (ANOVA) was conducted to determine statistical significance, followed by post-hoc multiple comparisons (Dunn's test) using SPSS 16.0 software (IBM Corporation, Armonk, NY). The value of $P< 0.05$ was considered significant.

Results

Effects of CSEI on cell apoptosis of K562 and CHRF cells. The results of Annexin V/PI staining showed that there were no differences on the rate of cell apoptosis in K562 and CHRF cells (Fig. S1).

Effects of CSEI on mice bone marrow leukocytes. After 3-week administration of CSEI or Tα1, the production of neutrophils, macrophages and B lymphocytes in mice bone marrow were analyzed by gating of CD45 via flow cytometric assay. $CD45^{+}Ly6G^{+}$ and $CD45^{+}CD19^{+}$, represent neutrophils and B lymphocytes, respectively. The numerical data showed that CTX significantly reduced the production of both the two kinds of leukocytes (P<0.01, Fig. S2). And 3-week treatment with CSEI and Tα1 attenuated this reduction (Fig. S2).

Effect of CSEI on neutrophils production in mice bone marrow. In order to better isolate myeloid populations when analyzing the neutrophils production in mice bone marrow, we detected the neutrophils by Mac-1(CD11b) and Ly6G, and $Mac\text{-}1^{+}Ly6G^{+}$ represents neutrophils. The production of neutrophils in model group was significantly decreased than that of control group (P<0.05), and the 3-week administration of CSEI enhanced the production of neutrophils in mice with CTX-induced hematopoietic injury by over 50% (P<0.01; Fig. S3).

Effect of CSEI on the number of HSCs and HPCs in mice bone marrow. The absolute number of bone marrow HSCs ($Lin^{-}c\text{-}kit^{+}sca\text{-}1^{+}$) and HPCs ($Lin^{-}c\text{-}kit^{+}sca\text{-}1^{-}$) were determined by flow cytometric assay. The results showed that CTX significantly reduced the number of HSCs and HPCs in bone marrow (P<0.001), which has been reversed to normal levels by CSEI at 2.25 mg/kg (P<0.01; Fig.S4). Additionally, CSEI at chosen doses significantly promoted the expression of HSCs and HPCs (P<0.05; Fig. S4).

Table list

Table S1. The regulatory effects of CSEI on cytokines and chemokines in spleen of mice

Coordinate	Target	Fold (vs. CTRL)			*P* (vs. Model)		
		Model	Tα1	CSEI	CTRL	Tα1	CSEI
B5,B6	G-CSF	0.78	1.13	1.47	0.107	0.116	0.024
B7,B8	GM-CSF	0.77	1.00	1.01	0.007	0.021	0.002
B9,B10	I-309	0.86	1.02	1.17	0.111	0.025	0.015
B11,B12	Eotaxin	0.78	0.79	1.19	0.482	0.958	0.366
B19,B20	IL-1β	0.70	0.98	1.27	0.014	0.009	0.002
B21,B22	IL-1ra	0.80	0.86	1.01	0.005	0.037	0.006
B23,B24	IL-2	0.77	1.25	1.48	0.188	0.065	0.021
C5,C6	IL-5	0.86	1.00	1.25	0.166	0.196	0.040
C7,C8	IL-6	0.79	0.89	1.10	0.071	0.028	0.001
C9,C10	IL-7	0.67	0.80	0.87	0.017	0.081	0.043
C11,C12	IL-10	0.75	0.88	0.98	0.060	0.453	0.090
C17,C18	IL-16	0.90	0.99	1.13	0.025	0.018	0.003
C19,C20	IL-17	0.75	1.02	1.13	0.086	0.060	0.038
C21,C22	IL-23	0.72	0.92	1.18	0.007	0.020	0.002
C23,C24	IL-27	0.64	1.07	1.39	0.045	0.025	0.006
D5,D6	KC	0.88	0.68	1.47	0.054	0.010	0.003
D7,D8	M-CSF	0.78	0.82	0.85	0.000	0.062	0.081
D9,D10	JE	1.04	0.93	1.70	0.021	0.037	0.000
D11,D12	MCP-5	1.07	1.11	1.03	0.010	0.213	0.034
D15,D16	MIP-1α	1.23	1.02	1.07	0.023	0.032	0.039
D19,D20	MIP-2	0.92	0.87	1.37	0.238	0.395	0.014
D21,D22	RANTES	0.91	1.10	1.24	0.059	0.095	0.019
D23,D24	SDF-1	1.05	1.21	1.29	0.105	0.079	0.032
E7,E8	TREM-1	0.91	0.95	1.06	0.043	0.102	0.008

Among 40 different kinds of cytokines evaluated using Mouse Cytokine Array Panel A Kit, 24 of which are significantly regulated by CSEI.

Figure list

Fig.S1. Effect of CSEI on cell apoptosis of K562 and CHRF. The K562 and CHRF cells were co-cultured with CSEI (0, 0.05, 0.1 mg/ml) for 24 h, and cell apoptosis of K562 and CHRF was detected by Annexin V/PI staining via flow cytometry (n=6).

Fig.S2. The numerical data of neutrophils (**a**) and B lymphocytes (**b**) expression in mice bone marrow cells after 3-week administration of CSEI or Tα1. Data are expressed as the means ± S.E.M. (n=10). $^{\#\#}P< 0.01$ and $^{\#\#\#}P< 0.001$ versus the control group, $^{*}P< 0.05$, $^{**}P< 0.01$ and $^{***}P< 0.001$ versus the model group.

Fig.S3. CSEI (2.25, 4.5 and 9 mg/kg) enhanced the CTX (100 mg/kg)-induced decrement of neutrophils production in murine bone marrow of mice after 3-week administration. (**a**) Flow cytometry analysis of the proportion of neutrophils in murine bone marrow, and Mac-1^{+}Ly6G^{+} represents neutrophils. (**b**) The numerical data are expressed as the means ± S.E.M. (n=10). $^{\#}P< 0.05$ versus the control group, $^{*}P< 0.05$, $^{**}P< 0.01$ and $^{***}P< 0.001$ versus the model group.

Fig.S4. The absolute number of HSCs (**a**) and HPCs (**b**) of per mouse (2 tibiae and 2 femora). The data are expressed as the means ± S.E.M. (n=10). $^{\#\#\#}P< 0.001$ versus the control group, $^{*}P< 0.05$, $^{**}P< 0.01$ and $^{***}P< 0.001$ versus the model group.

Fig.S5. The full-length blots of protein expression levels of P-RSK1p90, ELK1, c-Myc (**a**) and GATA-1, GATA-2 (**b**) in CHRF and K562 cells.

Fig.S6. The full-length blots of protein expression levels related to G-CSF-mediated JAK2/STAT3 signaling pathway in mice spleen (**a**) and the primary cultured BMNCs (**b**).

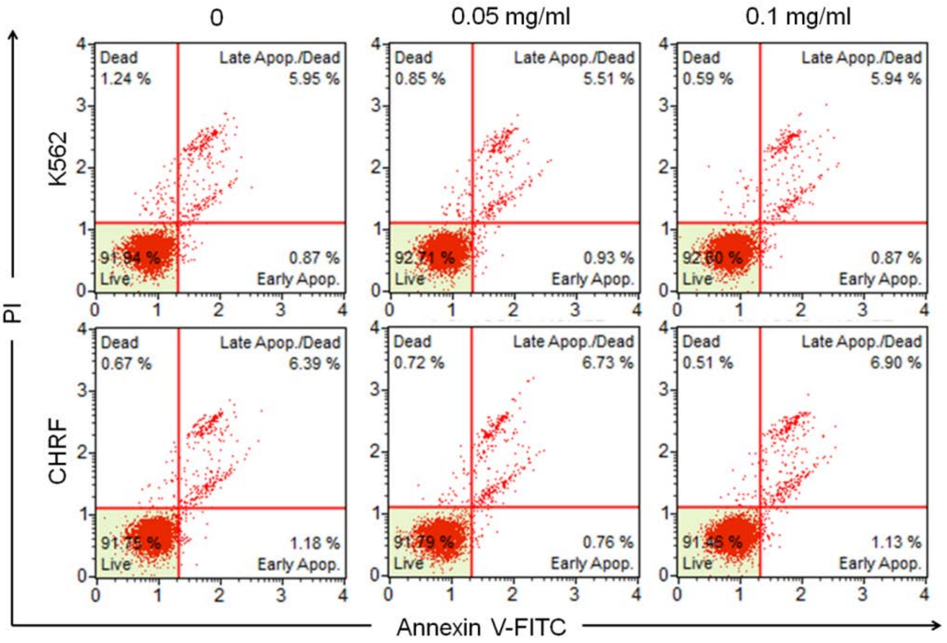
0
0.05 mg/ml
0.1 mg/ml
K562
CHRF
PI
Annexin V-FITC
Dead
1.24 %
Late Apop./Dead
5.95 %
91.94 %
Live
0.87 %
Early Apop.
Dead
0.85 %
Late Apop./Dead
5.51 %
92.71 %
Live
0.93 %
Early Apop.
Dead
0.59 %
Late Apop./Dead
5.94 %
92.60 %
Live
0.87 %
Early Apop.
Dead
0.67 %
Late Apop./Dead
6.39 %
91.75 %
Live
1.18 %
Early Apop.
Dead
0.72 %
Late Apop./Dead
6.73 %
91.79 %
Live
0.76 %
Early Apop.
Dead
0.51 %
Late Apop./Dead
6.90 %
91.46 %
Live
1.13 %
Early Apop.

Figure.S1

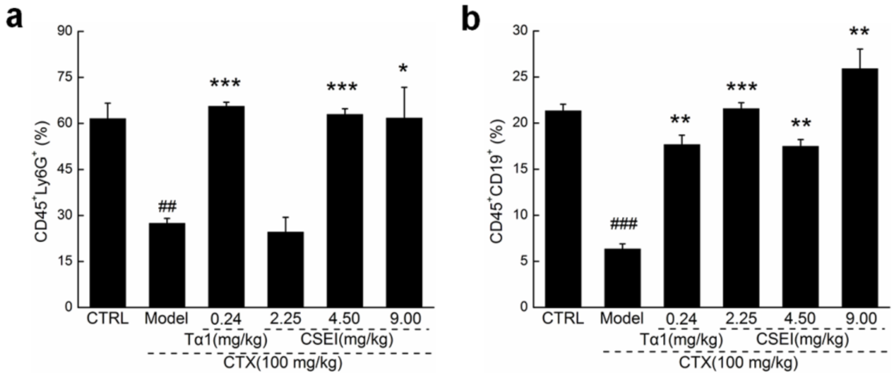
a
CD45+Ly6G+ (%)
90
75
60
45
30
15
0
##
***
***
*
CTRL
Model
0.24
2.25
4.50
9.00
Tα1(mg/kg)
CSEI(mg/kg)
CTX(100 mg/kg)
b
CD45+CD19+ (%)
30
25
20
15
10
5
0
###
**
***
**
**
CTRL
Model
0.24
2.25
4.50
9.00
Tα1(mg/kg)
CSEI(mg/kg)
CTX(100 mg/kg)

Figure.S2

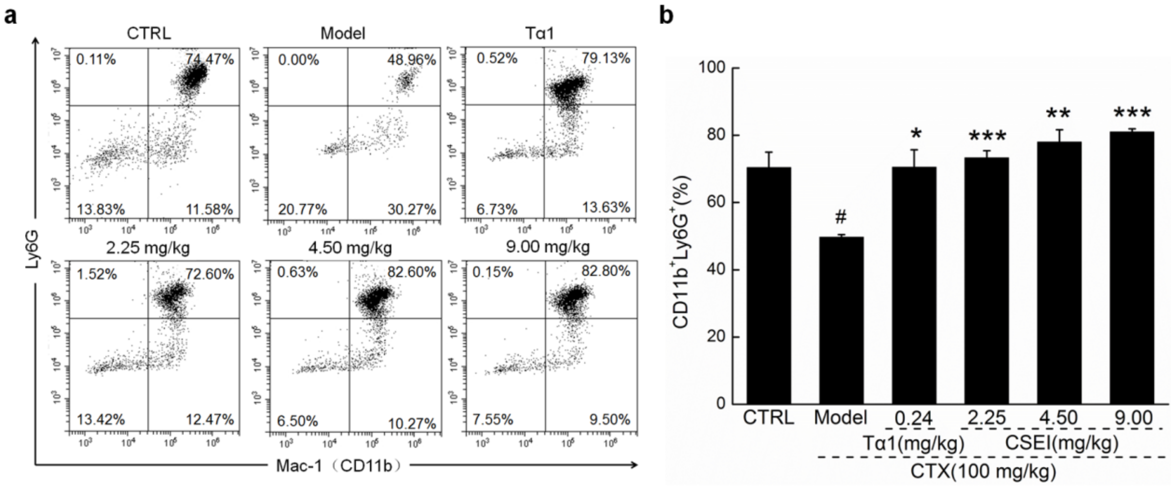
a
CTRL
Model
Tα1
0.11%
74.47%
13.83%
11.58%
0.00%
48.96%
20.77%
30.27%
0.52%
79.13%
6.73%
13.63%
2.25 mg/kg
4.50 mg/kg
9.00 mg/kg
1.52%
72.60%
13.42%
12.47%
0.63%
82.60%
6.50%
10.27%
0.15%
82.80%
7.55%
9.50%
Ly6G
Mac-1（CD11b）
b
CD11b⁺Ly6G⁺(%)
#
*
***
**
***
CTRL
Model
0.24
2.25
4.50
9.00
Tα1(mg/kg)
CSEI(mg/kg)
CTX(100 mg/kg)

Figure.S3

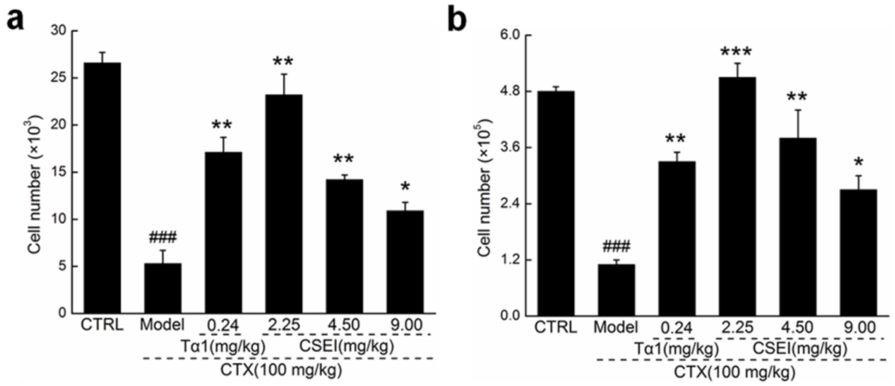
a
b
Cell number (×10³)
Cell number (×10⁵)
CTRL
Model
0.24
2.25
4.50
9.00
Tα1(mg/kg)
CSEI(mg/kg)
CTX(100 mg/kg)
###
**
***
*

Figure.S4

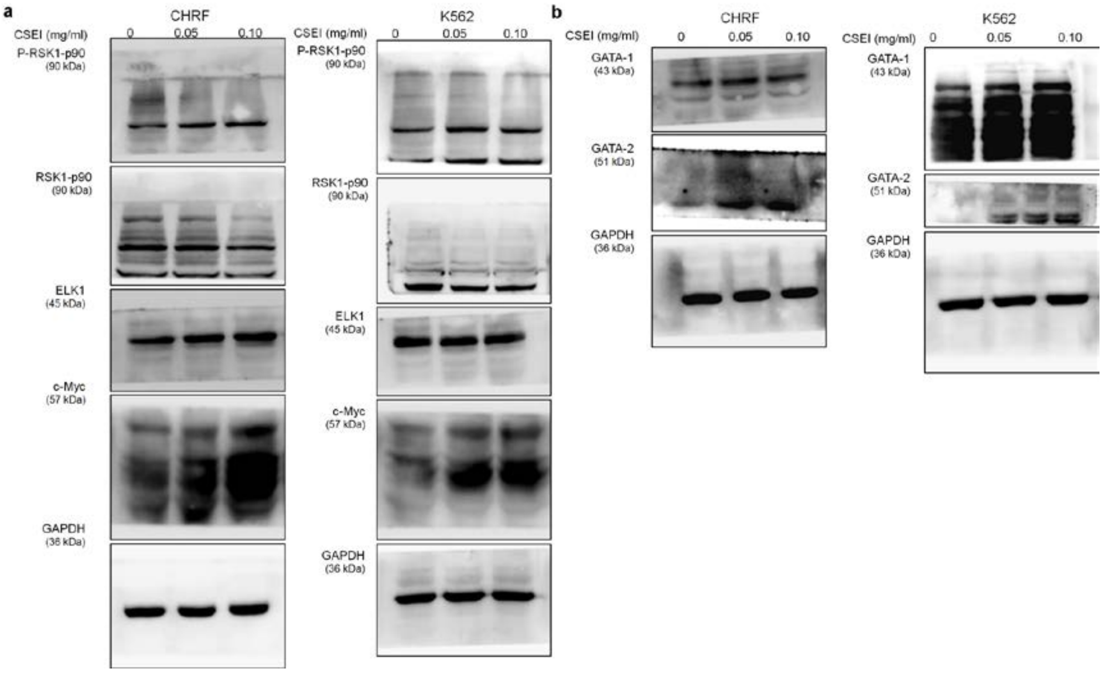
a
CHRF
CSEI (mg/ml) 0 0.05 0.10
P-RSK1-p90 (90 kDa)
RSK1-p90 (90 kDa)
ELK1 (45 kDa)
c-Myc (57 kDa)
GAPDH (36 kDa)
K562
CSEI (mg/ml) 0 0.05 0.10
P-RSK1-p90 (90 kDa)
RSK1-p90 (90 kDa)
ELK1 (45 kDa)
c-Myc (57 kDa)
GAPDH (36 kDa)
b
CHRF
CSEI (mg/ml) 0 0.05 0.10
GATA-1 (43 kDa)
GATA-2 (51 kDa)
GAPDH (36 kDa)
K562
CSEI (mg/ml) 0 0.05 0.10
GATA-1 (43 kDa)
GATA-2 (51 kDa)
GAPDH (36 kDa)

Figure.S5

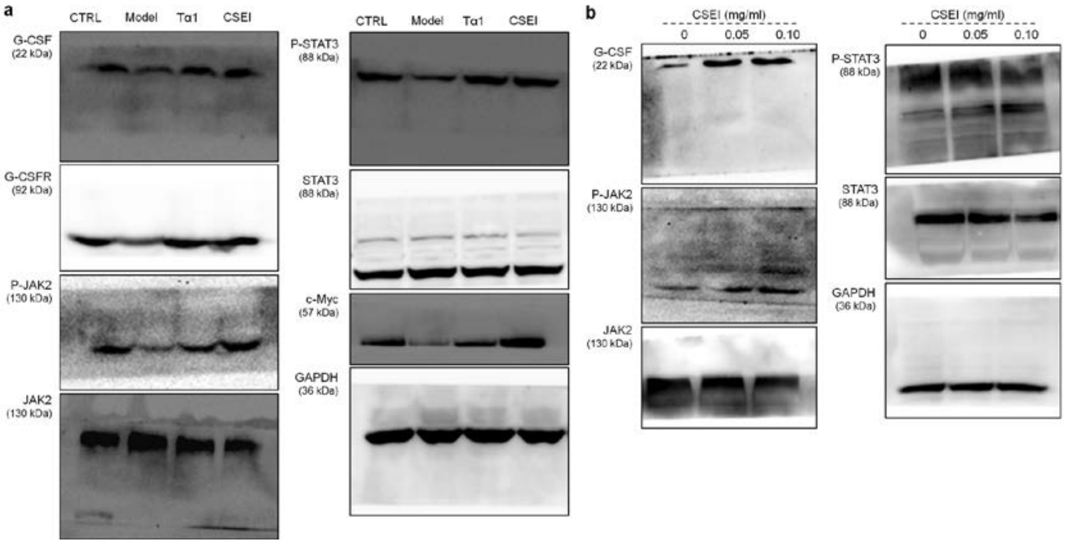
a
CTRL
Model
Tα1
CSEI
G-CSF
(22 kDa)
G-CSFR
(92 kDa)
P-JAK2
(130 kDa)
JAK2
(130 kDa)
P-STAT3
(88 kDa)
STAT3
(88 kDa)
c-Myc
(57 kDa)
GAPDH
(36 kDa)
b
CSEI (mg/ml)
0
0.05
0.10
G-CSF
(22 kDa)
P-JAK2
(130 kDa)
JAK2
(130 kDa)
P-STAT3
(88 kDa)
STAT3
(88 kDa)
GAPDH
(36 kDa)

Figure.S6
